# How Quantum is the Resonance Behavior in Vibrational Polariton Chemistry?

**DOI:** 10.1021/acs.jpclett.3c01154

**Published:** 2023-09-07

**Authors:** Marit R. Fiechter, Johan E. Runeson, Joseph E. Lawrence, Jeremy O. Richardson

**Affiliations:** † Department of Chemistry and Applied Biosciences, ETH Zürich, 8093 Zürich, Switzerland; ‡ Department of Chemistry, University of Oxford, Physical and Theoretical Chemistry Laboratory, South Parks Road, Oxford OX1 3QZ, United Kingdom

## Abstract

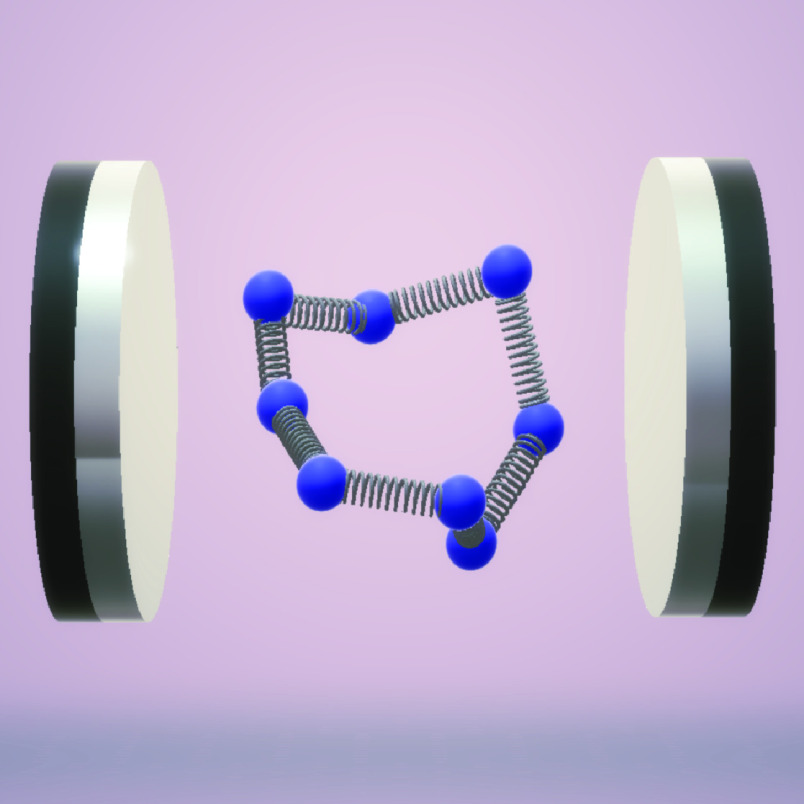

Recent experiments in polariton chemistry have demonstrated that reaction rates can be modified by vibrational strong coupling to an optical cavity mode. Importantly, this modification occurs only when the frequency of the cavity mode is tuned to closely match a molecular vibrational frequency. This sharp resonance behavior has proved to be difficult to capture theoretically. Only recently did Lindoy et al. [Nat. Commun.2023, 14, 27333717329910.1038/s41467-023-38368-xPMC10182063] report the first instance of a sharp resonant effect in the cavity-modified rate simulated in a model system using exact quantum dynamics. We investigate the same model system with a different method, ring-polymer molecular dynamics (RPMD), which captures quantum statistics but treats dynamics classically. We find that RPMD does not reproduce this sharp resonant feature at the well frequency, and we discuss the implications of this finding for future studies of vibrational polariton chemistry.

A recent series of experiments has revealed the surprising result that one can alter chemical reaction rates just by placing the reaction mixture in an optical cavity,^1−9^ i.e., between a pair of carefully spaced mirrors which support standing waves of light at specific frequencies. In particular, when a cavity mode is strongly coupled to molecular vibrations (called vibrational strong coupling, or VSC),^10,11^ the rate constant of ground-state reactions can be modified even without external driving, i.e., without explicitly adding photons into the cavity. As the cavity mode can be treated as a harmonic oscillator coupled to the molecular system under study, it is relatively straightforward to incorporate into standard theoretical chemistry methods. However, in spite of the plethora of theoretical studies conducted on the topic (recently reviewed in, e.g., refs (12)–^14^), the mechanism behind this cavity effect on the chemical reaction rate is not yet well-understood.

One of the features observed in experiments that has proven hard to reproduce theoretically is the resonance behavior: the rate-constant modification is only significant when the cavity length is tuned such that one of the cavity modes is in resonance with a vibrational mode in the reaction mixture (either of a reactant^2−4,6−9^ or a solvent molecule^4,5,9^). The width of this resonant feature in the plot of rate versus cavity frequency is comparable to the line width of the molecular resonance in the infrared spectrum,^2,3,5,7^ typically on the order of tens of wavenumbers. Another feature of experiments complicating a theoretical analysis is the fact that in experiments a large number of molecules are coupled to a single cavity mode. This induces collective effects, so that spectral characteristics such as the Rabi splitting depend on the number of molecules coupled to the cavity.^15,16^ The question remains as to the mechanism by which these collective effects influence the rate. However, for now we will constrain ourselves to the single-molecule regime, as the focus of this work is to compare an approximate theory to a fully quantum-mechanical benchmark, for which the extension to multiple molecules quickly becomes prohibitively expensive.

In this work, we focus on the resonance behavior and further investigate to what extent quantum effects could play a role for these sharp resonances in the rate around a reactant vibrational frequency. Although analytical rate theories such as Grote–Hynes theory,^17^ Eyring theory,^18^ or Pollak–Grabert–Hänggi theory^19^ do predict a small cavity effect, these effects tend to be spread over a broad range of cavity frequencies. Additionally, the largest effect observed in these theories does not typically occur when a cavity mode is on resonance with a vibrational mode of the reactant, contrary to experimental observations. Only recently has a sharp resonance behavior, more in line with experiment, been reported theoretically by Lindoy et al.^20^ In that study, they used a fully quantum-dynamical approach (hierarchical equations of motion, HEOM)^21^ for a specific low-friction parameter regime of a model double-well system coupled to a cavity mode. If the cavity is lossy, these quantum simulations give peaks in the rate modification centered at the reactant frequency with a full width at half-maximum (fwhm) as small as 80 cm^–1^, which is significantly narrower than the fwhm seen in earlier classical simulations^22,23^ (e.g., ∼350 cm^–1^ for the isomerization of HONO^22^). Importantly, the quantum results are comparable to the resonance widths observed in experiment,^2,3,5,9^ although it should be noted that Lindoy et al. considered only a single molecule in the cavity, making up for the lack of collective enhancement of the effect by choosing a large effective light–matter coupling strength.

We assess the importance of quantum effects in forming these sharp peaks by comparing the HEOM results with ring-polymer molecular dynamics (RPMD) rate theory.^24−26^ RPMD is based on imaginary-time path integrals and is thus able to capture quantum statistics (including tunneling and zero-point energy effects) but treats dynamics classically and contains no phase information, meaning it cannot reproduce effects due to real-time quantum coherences or quantization of vibrational states.^27^ Hence, whether RPMD succeeds in reproducing the HEOM results gives us insight into whether the quantum effects responsible for the sharp resonant feature are statistical or dynamical in nature. This will also have important implications for future studies in this field: as quantum-dynamics calculations on full-dimensional realistic systems are prohibitively expensive, it is natural to resort to more affordable methods that capture nuclear quantum effects only approximately or neglect them entirely.^22,28,29^ The results in this study show that to reproduce the sharp resonances seen in the low-friction models studied in ref (20), classical dynamics or even RPMD simulations will not suffice: one needs to treat (at least part of) the problem quantum-mechanically.

*Model.* The model system we study here is equivalent to that of ref (20). The Hamiltonian can be expressed as
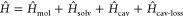
1where *Ĥ*_mol_ is the molecular Hamiltonian, *Ĥ*_solv_ describes the solvent and its coupling to the molecule, *Ĥ*_cav_ describes the cavity mode and its interaction with the molecule, and *Ĥ*_cav-loss_ represents cavity loss through interaction with electromagnetic modes outside the cavity. A diagrammatic representation of the degrees of freedom in this Hamiltonian and their coupling is shown in Figure 1a. All calculations are performed at a temperature of 300 K.

**Figure 1:**
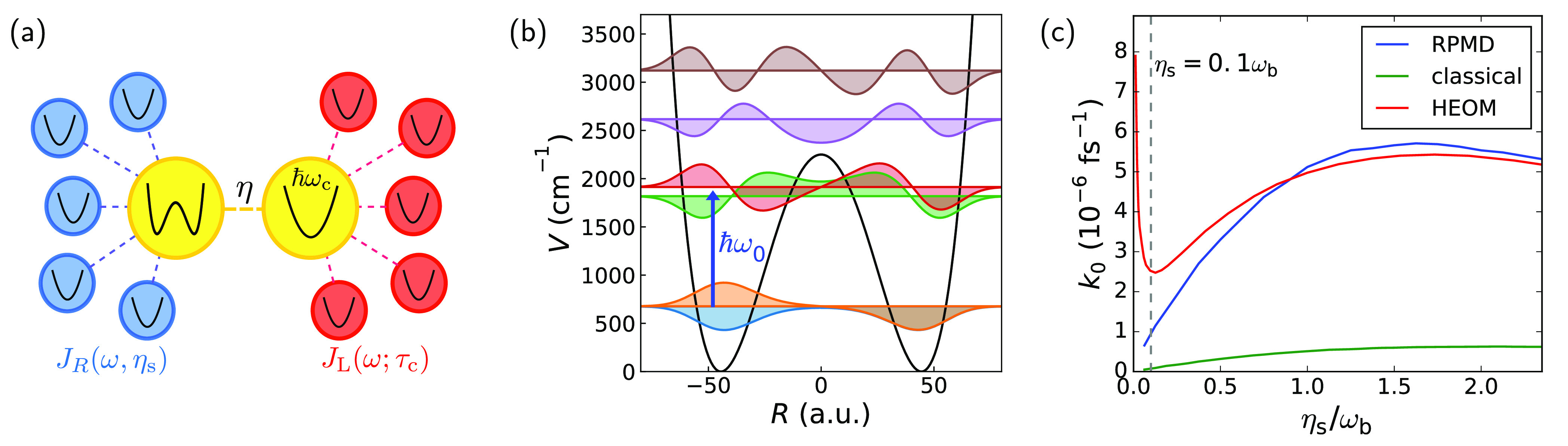
Outline of the model. (a) Diagram representing the degrees of freedom in the model and their coupling. The molecule–cavity coupling strength is given by η, the magnitude of the solvent friction is determined by η_s_ (blue), and the strength of the coupling between the cavity mode and external modes is given by τ_c_ (red). (b) Double well I and its vibrational eigenstates, with the blue arrow indicating the well frequency ω_0_. (c) Rate constant *k*_0_ as a function of solvent friction η_s_ outside the cavity (i.e., η = 0) for system I. The gray dashed line indicates the friction at which the results of Figure 2 were obtained.

The molecular Hamiltonian *Ĥ*_mol_ is chosen to be a one-dimensional symmetric double well, so that for the molecular coordinate *R* (with unit mass) we have *Ĥ*_mol_ = *P̂*_*R*_^2^/2 + *V*(*R̂*), with the potential given by
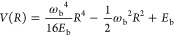
2where ω_b_ is the imaginary part of the barrier frequency and *E*_b_ is the barrier height of the double well. Note that, as illustrated in Figure 1b, the vibrational states of such a double-well system are split by a very small energy, the tunneling splitting. We define the (anharmonic) well frequency as the difference between the first and second pairs of these tunneling-split states, i.e., , where *E*_*n*_ denotes the energy of the *n*th eigenstate of *Ĥ*_mol_. In this work we study three of these double-well systems: (I) a relatively shallow double-well system (studied in the main text of ref (20)), with ω_b_ = 1000 cm^–1^ and *E*_b_ = 2250 cm^–1^, so that ω_0_ ≈ 1190 cm^–1^; (II) a double-well system with a lower well frequency (studied in the Supporting Information of ref (20)), with ω_b_ = 500 cm^–1^ and *E*_b_ = 2000 cm^–1^, yielding ω_0_ ≈ 650 cm^–1^; and (III) a double well that is deeper than system I but retains a comparably high well frequency (also studied in the Supporting Information of ref (20)), with ω_b_ = 1000 cm^–1^ and *E*_b_ = 3000 cm^–1^, yielding ω_0_ ≈ 1250 cm^–1^.

The interaction of the molecule with the solvent is taken to be
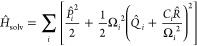
3which couples the molecular coordinate to a harmonic bath described by the canonical operators *P̂*_*i*_ and *Q̂*_*i*_. Here we take this bath to be characterized by a Debye spectral density
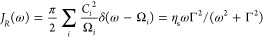
4 where we set Γ = 200 cm^–1^; η_s_ can be varied to change the solvent friction.

Within the dipole approximation, the coupling of the molecular coordinate to the cavity mode is given by the Pauli–Fierz Hamiltonian:^12,13^
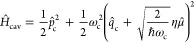
5where *p̂*_c_ and *q̂*_c_ are the canonical displacement field operators, respectively; ω_c_ is the cavity frequency;  is a measure of the coupling strength, where ϵ_0_ is the permittivity of vacuum and  is the quantization volume; and μ̂ is the dipole moment operator projected onto the electronic ground state and along the cavity polarization (μ̂ = **ê**·**μ̂**). We follow ref (20) and choose the dipole moment to be linear in the molecular coordinate (μ̂ = *R̂*).

If the cavity mirrors are not perfectly reflective, the electromagnetic mode inside the cavity can interact with the continuum of modes outside the cavity, enabling, for example, the escape of a photon from the cavity. This can effectively be described by
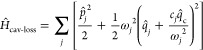
6where *p̂_j_* and *q̂*_*j*_ are the canonical operators associated with field modes outside the cavity. Making the assumption that this bath of external light modes is Markovian, one can relate the parameters in this Hamiltonian, i.e., the set of frequencies ω_*j*_ and couplings *c*_*j*_ described by a spectral density , to the cavity lifetime τ_c_. One possible such relation is given in ref (20):
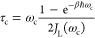
7which is the definition we will use throughout this work for consistency.^100^ In ref (20), *J*_L_(ω) was chosen to be a Debye spectral density *J*_L_(ω) = η_L_ωΓ_L_^2^/(ω^2^ + Γ_L_^2^) with Γ_L_ = 1000 cm^–1^. Debye baths are the natural choice for HEOM calculations, whereas for RPMD there is a significant advantage in using an Ohmic spectral density instead. Therefore, to reduce the cost of our RPMD calculations for some calculations (see Table 1 in the Supporting Information), we replaced this Debye spectral density by an Ohmic spectral density *J*_L_(ω) = γ_L_ω with γ_L_ defined by τ_c_ and eq 7. As the spectral density near ω_c_ is unaltered, this is not expected to significantly change the dynamics. Details of our treatment of the spectral densities and results supporting the validity of this exchange are given in the Supporting Information.

*Theory.* We perform the calculations in this study with RPMD.^24,25,31,32^ Here we summarize the general idea behind this method; for details of our implementation, we refer the reader to the Supporting Information.

In short, RPMD is based on discretized closed paths in imaginary time called ring polymers. These ring polymers emerge for example in path-integral molecular dynamics,^33,34^ where by sampling over ring-polymer configurations one can compute static equilibrium properties of a quantum system exactly. It is, however, not feasible to rigorously extend this to real-time dynamics because of the infamous sign problem. RPMD circumvents this by instead propagating the ring polymer classically to obtain an approximation to real-time quantum correlation functions such as the flux–side correlation function from which one can extract the rate constant. RPMD is exact at short times for correlation functions involving functions of position. On this basis, RPMD rate theory can be shown to be accurate for reaction rates that are determined by a free-energy bottleneck and to capture the effects of zero-point energy and tunneling on the rate.^24−26,35,36^ On the other hand, it is not able to capture truly quantum-dynamical effects, such as interference or effects involving vibrational quantization beyond those encapsulated by the zero-point energy.^27^ Therefore, whether or not RPMD captures the resonance behavior observed with quantum dynamic simulations in ref (20) will elucidate the relative importance of these static and dynamical quantum effects.

*System I: Shallow Wells with High Frequency.* We start by investigating the behavior of the chemical reaction rate without the cavity, *k*_0_, with increasing solvent friction, η_s_, in Figure 1c. For frictions larger than η_s_ ≈ 0.2ω_b_, this figure shows typical Kramers turnover behavior,^24,37^ i.e., the rate increases with increasing friction until it reaches a maximum (“turnover”) and then decreases with increasing friction after that. We see that RPMD is most accurate for friction strengths close to the turnover and higher, where it is within a few percent of the quantum result. As one approaches lower frictions, RPMD is less accurate at predicting the rate. One possible reason for this is coherent nuclear tunneling between the wells, indicated by the sharp rate profile for very low friction, which is a fundamentally quantum-dynamical effect not captured by RPMD. Another possibility is that the frequency ω_0_ is too large for the vibrational energy transfer between the molecule and the bath to be well-described by classical mechanics,^38^ which in turn affects the reaction rate because in the low-friction regime the overall rate is limited by the diffusion of energy between the bath and the reaction coordinate. Note that while the RPMD results are not perfect, they are significantly better than the classical rate prediction, which underestimates the quantum benchmark rate by about an order of magnitude over the entire range of frictions considered.

In Figure 2 we investigate the change in rate, *k*/*k*_0_, when the molecular coordinate is coupled to the cavity mode for a low value of the solvent friction, η_s_ = 0.1ω_b_. In particular, in ref (20) it was found that the effect of the cavity is negligible when the cavity is lossless (τ_c_ → *∞*). The authors explained this by pointing out that in this case, energy transfer from the molecular mode to the cavity mode may be possible, but from there the energy cannot dissipate. The cavity mode will therefore not efficiently dampen the motion in the reaction coordinate. When the coupling between the cavity mode and the environment is increased (i.e., τ_c_ is reduced), a dramatic increase in the exact rate is observed. Moreover, the resulting rate profile as a function of cavity frequency features a single sharp peak (with a fwhm of about 80 cm^–1^ for τ_c_ = 100 fs cavity lifetime), and it is centered around the well frequency. In this respect, it is in agreement with experimental observations (although the experiments involve many molecules in a cavity).

**Figure 2:**
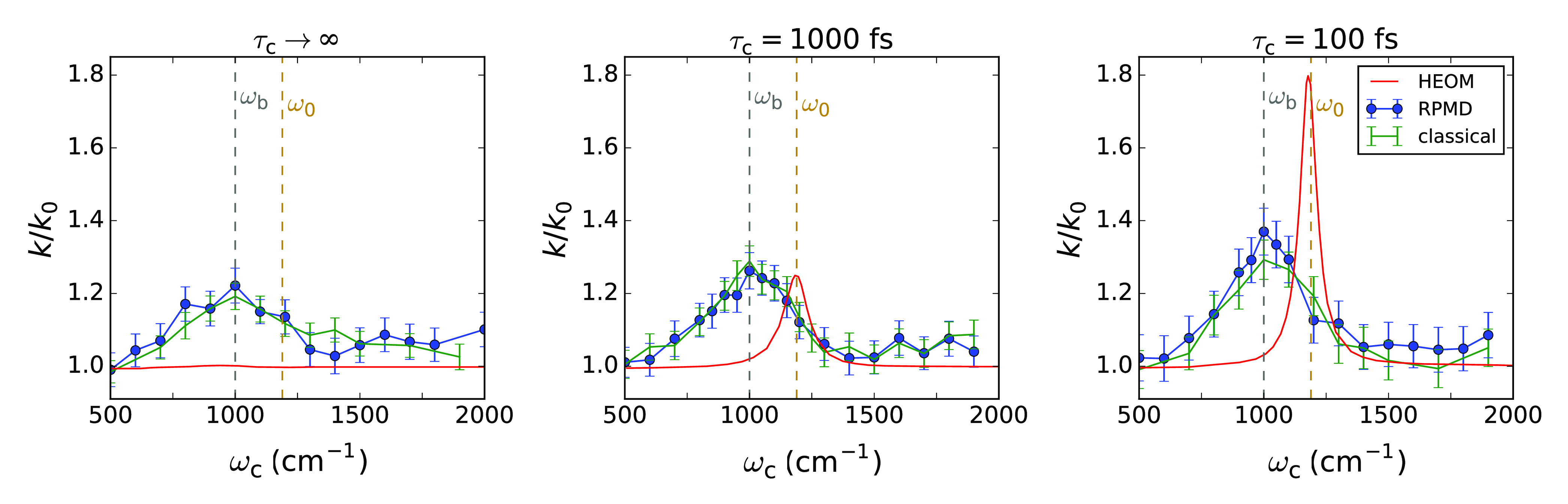
Cavity modification of the rate of system I as a function of cavity frequency ω_c_ for a set of cavity lifetimes τ_c_, with η = 0.00125 a.u. and η_s_ = 0.1ω_b_. Neither classical dynamics nor RPMD is able to reproduce the sharp peak in rate enhancement at the well frequency, ω_0_; instead, they give a broad peak centered at the barrier frequency ω_b_. The error bars indicate a 68% confidence interval.

Both classical and RPMD simulations display markedly different behavior from the HEOM results: the peak in the cavity-induced rate enhancement is centered around the barrier frequency ω_b_ rather than the well frequency, and it is also a much broader feature, its fwhm spanning hundreds of wavenumbers. Interestingly, the effect of cavity loss (τ_c_) on the rate is much smaller here: the rate modification is not entirely suppressed for lossless cavities, as it was in the quantum case, and the results do not change dramatically when introducing a finite cavity lifetime (from a rate enhancement by a factor of 1.2 for a lossless cavity to a factor of 1.4 for a cavity with a lifetime of τ_c_ = 100 fs).

The large difference between the classical simulation and the HEOM results indicates that quantum-mechanical effects are important. Interestingly, even though the absolute rate without cavity, *k*_0_, predicted by classical and RPMD simulations differs by a factor of about 8, they predict similar rate modifications, *k*/*k*_0_, which agree with each other within the error bars. This implies that changes to the quantum statistics are not the dominant factor in the effect the cavity has on the relative rate *k*/*k*_0_ in this case. Instead, the discrepancy between both the classical and RPMD results and the HEOM results indicates that quantum dynamics plays a key role. What is unclear from these results, however, is whether the quantum-dynamical effect that RPMD is lacking is primarily coherent tunneling between the wells or if it is a result of quantum-mechanical effects on the rate of energy transfer to or from the double-well system. In order to assess this, it is necessary to consider systems with smaller tunneling splittings, for which the coherent tunneling is suppressed.

*System II: Shallow Wells with Low Frequency.* We now move on to a double-well system with a broader barrier and lower well frequency, so that there are three (instead of two) tunneling-split eigenstates below the barrier top (see Figure 3a; cf. Figure 1b). The cavityless rate constant as a function of solvent friction η_s_, shown in Figure 3b, reveals that this system loosely speaking behaves less quantum-mechanically; there is, for example, no coherent-tunneling regime at very low friction. The classical rate in this case only underestimates the full quantum rate constant by a factor of 2, whereas RPMD yields a spot-on prediction of the rate. This is in line with the hypothesis^39^ that the rate-limiting step is the rate of vibrational energy transfer between the reactive mode and the bath: it is well-known that this is well-described with classical dynamics for low-frequency system modes (such as in system II), while the classical description breaks down and a quantum treatment becomes necessary for high-frequency system modes (such as in system I).

**Figure 3:**
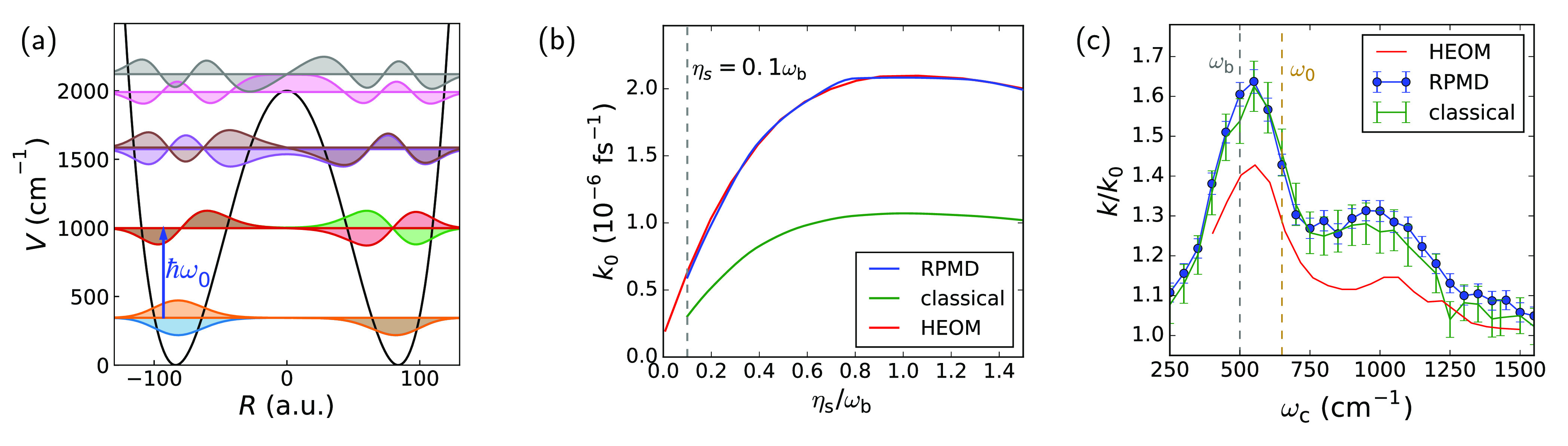
System II and cavity modification of its rate. (a) Double well II and its eigenstates. The blue arrow indicates the well frequency ω_0_. (b) Rate constant *k*_0_ as a function of solvent friction η_s_ outside the cavity (η = 0). (c) Rate-constant modification *k*/*k*_0_ as a function of cavity frequency for η_s_ = 0.1ω_b_, η = 0.005 a.u., and τ_c_ = 1000 fs.

Coupling the cavity mode to this low-frequency double well yields quite a different cavity-frequency dependence of the rate, as displayed in Figure 3c. First, the rate profile is not composed of a single sharp peak; rather, it is made up of a much broader peak (fwhm > 300 cm^–1^) and a somewhat lower broad shoulder. Moreover, the main peak is no longer centered at the molecular vibrational frequency; at ω_c_ ≈ 560 cm^–1^ it is somewhere between the barrier frequency (ω_b_ = 500 cm^–1^) and the well frequency (ω_0_ ≈ 650 cm^–1^). This is qualitatively similar to what is known about the frequency dependence in the classical depopulation factor in Pollak–Grabert–Hänggi theory, as studied in ref (19).

Interestingly, in this case the rate-enhancement profile is captured qualitatively by the classical simulations, and adding in quantum statistics via RPMD has little additional effect. This suggests that the origin of the cavity-induced rate modification in this system can be understood classically. However, although RPMD agrees very well with HEOM in the cavityless case, it slightly overestimates the magnitude of the rate modification by the cavity. This is in line with the saturation of the HEOM rate modification for larger light–matter coupling strengths, η, observed in the Supporting Information of ref (20). This saturation effect is much less pronounced in the classical case, and RPMD does not improve classical dynamics in capturing this. Nevertheless, this effect is relatively small, and accounting for the improvement in accuracy of the *k*_0_ results from RPMD compared to classical mechanics, one can conclude that RPMD does rather well in this system. Note, however, that by going from system I to system II, we have also lost the sharp peak centered at ω_0_ in the HEOM results, and therefore, this system does not exhibit one of the key features of the experimental results. It therefore remains to be seen whether RPMD can accurately describe systems in which coherent tunneling is diminished but for which the resonance behavior in the presence of a cavity persists. This is what we assess next.

*System III: Deep Wells with High Frequency.* The last system we consider (Figure 4a) has a higher barrier than system I and a higher frequency than system II, comparable to that of system I (ω_0_ ≈ 1250 cm^–1^). This means that while coherent tunneling processes like that in system I are suppressed, we expect that vibrational energy transfer may (still) be quantum-dynamical in character. This is in contrast to the more classical system II, where RPMD turned out to be sufficient for predicting the cavityless rate.

**Figure 4:**
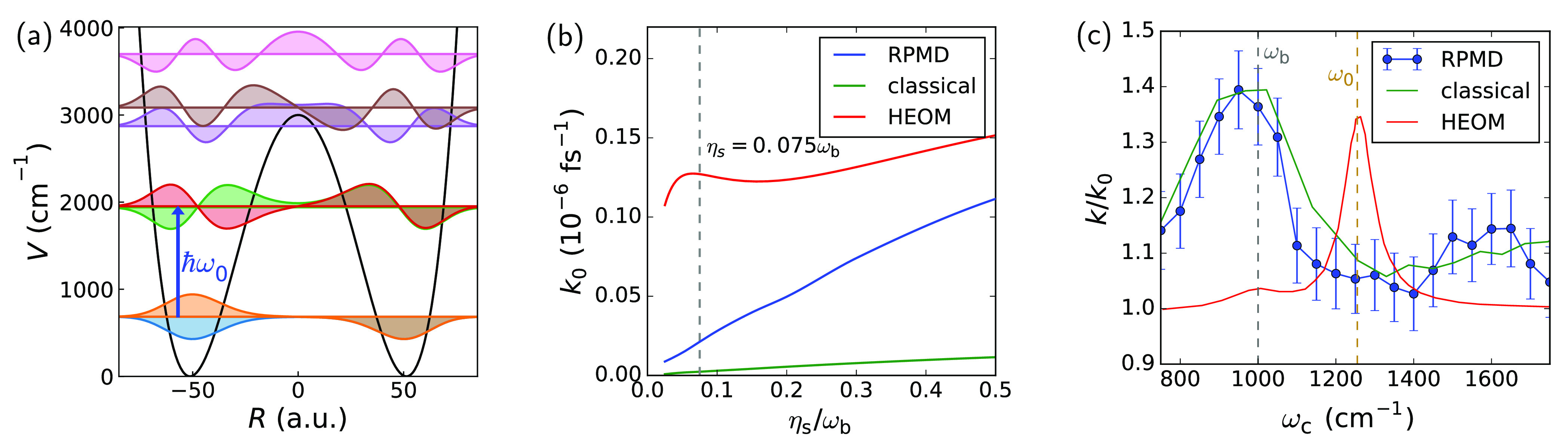
System III and cavity modification of its rate. (a) Double well III and its eigenstates. The blue arrow indicates the well frequency ω_0_. (b) Rate constant *k*_0_ as a function of solvent friction η_s_ outside the cavity (η = 0). (c) Rate-constant modification *k*/*k*_0_ as a function of cavity frequency for η_s_ = 0.075ω_b_, η = 0.00125 a.u., and τ_c_ = 100 fs.

Indeed, from the Kramers curve shown in Figure 4b, it is clear that for the range of solvent frictions considered, the rate is dominated by dynamical quantum effects that cannot be captured by RPMD: although RPMD starts approaching the HEOM results for higher friction, it still underestimates the rate by ∼26% for the highest friction plotted. Note that this is well before Kramers turnover; it is likely that RPMD reaches reasonable agreement with the HEOM around turnover, as it did for system I.

The effect of coupling the cavity to this double well is shown in Figure 4c. As for system I, the HEOM results show a quite sharp resonant rate enhancement at the well frequency, while again the classical and RPMD results fail to capture this and instead produce a broader feature around the barrier frequency. The implications of this finding are discussed below.

*Discussion.* In summary, we have investigated three variations of the model system studied in ref (20). In system I, where the molecular coordinate is a relatively shallow and high-frequency double well, full quantum dynamics predicts that the rate modification as a function of cavity frequency peaks sharply around the well frequency. We show that this feature cannot be reproduced with classical dynamics and that even adding quantum statistics with RPMD does not improve upon this. This indicates that producing the resonance in this system requires quantum-dynamical effects. In system II we consider a deeper double-well system with a lower well frequency, where quantum effects should play a less prominent role. Indeed, even classical dynamics qualitatively captures the correct frequency-dependent rate modification *k*/*k*_0_ in this case (even though one needs statistical quantum effects in RPMD to correctly predict the absolute rate constant). This system, however, lacks the sharp resonance feature that makes system I so intriguing. Finally, in system III we consider a double well that is deeper than that of system I, so that one would expect the role of coherent tunneling processes to be diminished, but still has a high frequency comparable to that of system I, meaning vibrational energy transfer is expected to have a quantum-mechanical character. This double well again exhibits a sharp resonance about the well frequency in the rate enhancement, and again neither RPMD nor classical simulations can capture this. These findings combined suggest that the rate-controlling dynamical quantum effects in question involve the transfer of vibrational energy into the reactive molecular mode; it is likely this process that is modified by the cavity. This is also supported by a very recent study^40^ in which it was shown that a resonant cavity can enhance the steady-state population of the reactive mode.

We note in the passing that one may be able to capture vibrational energy transfer processes correctly with Matsubara dynamics.^41^ Matsubara dynamics is formally the most accurate way of combining quantum statistics with classical dynamics. However, it does so at the expense of introducing a highly oscillatory phase, rendering it impractical and in fact more expensive to perform than exact quantum dynamics. Although the first converged Matsubara calculations for a Morse oscillator strongly coupled to a harmonic bath have recently been reported,^42^ the weak system–bath coupling regime is still out of reach. We can therefore not exclude the possibility that Matsubara dynamics would produce the correct results for this problem. We have shown, however, that classical dynamics *without phase factors* does not suffice.

The question remains as to whether the mechanism at play in these models is the same as the mechanism causing the rate modification in experiment. First, modification of the rate of vibrational energy transfer will only significantly affect the overall rate if it is the rate-limiting step, i.e., for reactions in the low friction regime. Chemical reactions typical of this regime are unimolecular dissociation reactions in low-pressure gases,^37^ although instances of energy-diffusion-limited reactions in solution have also been reported.^43,44^ Further investigations are needed to reveal whether the model studied here is representative of the (solution-phase) reactions performed in experiment.

Additionally, it should be assessed whether this effect survives when the number of molecules in the cavity increases. In particular, it would be interesting to investigate whether one can simply recover the effects of collectivity in a single-molecule simulation by rescaling the results. This simple relation between single- and many-molecule results is not a given: for example, it has been suggested that coupling more molecules to the cavity may also have the effect of “sharpening up” the peak and moving its position.^9^ In this light, one may not want to abandon high-friction models altogether.

In any case, if it turns out that the reactions studied in experiment are indeed in the energy-diffusion-limited regime, then our results demonstrate that quantum dynamics is vital for capturing the single-molecule resonance behavior correctly (as the lion’s share of molecular vibrations are “high frequency” in the context of vibrational energy transfer^45^), and one cannot get away with doing classical molecular dynamics (as in refs (22) and (29)) or even RPMD, as this will yield qualitatively different results. However, not all is lost: one may for example still be able to cut computational costs by only treating important molecular and cavity degrees of freedom quantum-mechanically in a mixed quantum–classical scheme.^46−55^ This is another exciting avenue for further exploration.

## Supporting Information Available

The following files are available free of charge: The Supporting Information is available free of charge at https://pubs.acs.org/doi/10.1021/acs.jpclett.3c01154.

Details on the computational method used and results supporting the validity of the replacement of the Debye spectral density by an Ohmic spectral density in the light-mode bath (PDF)
