# Supplementary material for: How Quantum is the Resonance Behavior in Vibrational Polariton Chemistry?
Source: J Phys Chem Lett. 2023 Sep 7;14(36):8261–7. doi: 10.1021/acs.jpclett.3c01154 (PMC10510439; doi:10.1021/acs.jpclett.3c01154)
Supplement: Supplementary file 1 — jz3c01154_si_001.pdf [file jz3c01154_si_001.pdf]

Supporting Information: How Quantum is the Resonance Behavior in Vibrational Polariton Chemistry?

Marit R. Fiechter,† Johan E. Runeson,‡ Joseph E. Lawrence,† and Jeremy O. Richardson*,†

†*Department of Chemistry and Applied Biosciences, ETH Zürich, 8093 Zürich, Switzerland*

‡*Department of Chemistry, University of Oxford, Physical and Theoretical Chemistry Laboratory, South Parks Road,Oxford, OX1 3QZ, United Kingdom*

E-mail: jeremy.richardson@phys.chem.ethz.ch

1 Computational details

1.1 RPMD rate theory

The RPMD approximation to the rate constant, k, is defined via the flux–side correlation function:

$$C_{\mathrm{fs}}(t) = \frac{1}{(2\pi\hbar)^{Nf}} \iint \mathrm{d}^{Nf}\mathbf{p}\, \mathrm{d}^{Nf}\mathbf{q}\, \mathrm{e}^{-\beta_N H_N(\mathbf{p},\mathbf{q})}\, F(\mathbf{p},\mathbf{q})\, \theta[s(\mathbf{q}(t))], \tag{S1}$$

where N is the number of ring-polymer beads, $\beta = 1/k_{\mathrm{B}}T$ is the inverse temperature, $\beta_N = \beta/N$, f is the number of physical degrees of freedom, $\theta(\cdot)$ is the Heaviside step function, and $s(\mathbf{q})$ defines the dividing surface; in this work, we use a dividing surface based on the centroid of the ring polymer, $\sum_i \mathbf{q}_i/N$. Note that $\mathbf{q}(t)$ in Eq. S1 is obtained by classically evolving $\mathbf{q}$ forward in time according to Hamilton's equations of motion generated by the N-bead ring-polymer Hamiltonian,

$$H_N(\mathbf{p},\mathbf{q}) = \sum_{i=1}^{N} \left[\frac{\mathbf{p}_i^2}{2m} + \frac{m}{2\beta_N^2\hbar^2}(\mathbf{q}_i - \mathbf{q}_{i+1})^2 + V(\mathbf{q}_i) \right]. \tag{S2}$$

The flux through the dividing surface is given by

$$F(\mathbf{p},\mathbf{q}) = \dot{\theta}[s(\mathbf{q})] = \delta[s(\mathbf{q})] \sum_{i=1}^{N} \sum_{\nu=1}^{f} \frac{\partial s(\mathbf{q})}{\partial q_{i,\nu}} \frac{p_{i,\nu}}{m}. \tag{S3}$$

Using this, we can calculate the rate constant by converging the expression of the rate:[1]

$$k = \lim_{t\to\infty} \frac{Q_\mathrm{r}^{-1}\, C_\mathrm{fs}(t)}{1 - (Q_\mathrm{r}^{-1} + Q_\mathrm{p}^{-1}) \int_0^t \mathrm{d}\tau\, C_\mathrm{fs}(\tau)}. \tag{S4}$$

In practice, the $t \to \infty$ limit means that one needs to run the simulation for long enough such that the right-hand side of Eq. S4 reaches a plateau, *i.e.* does not change considerably when further increasing the length of the simulation. Finally, Q_r and Q_p are the reactant and product partition functions, defined by

$$\begin{aligned} Q_\mathrm{r} &= \frac{1}{(2\pi\hbar)^{Nf}} \iint \mathrm{d}^{Nf}\mathbf{p}\, \mathrm{d}^{Nf}\mathbf{q}\, \mathrm{e}^{-\beta_N H_N(\mathbf{p},\mathbf{q})}\, \theta[-s(\mathbf{q})], \\ Q_\mathrm{p} &= \frac{1}{(2\pi\hbar)^{Nf}} \iint \mathrm{d}^{Nf}\mathbf{p}\, \mathrm{d}^{Nf}\mathbf{q}\, \mathrm{e}^{-\beta_N H_N(\mathbf{p},\mathbf{q})}\, \theta[s(\mathbf{q})]. \end{aligned} \tag{S5}$$

For the symmetric systems we study in this work, $Q_\mathrm{r} = Q_\mathrm{p}$.

The RPMD rate constant is obtained in the $N \to \infty$ limit, whereas for $N = 1$, the above equations reduce to classical rate theory.

1.2 Explicit and implicit baths

In our system we have two harmonic baths, which are characterized by their spectral densities. Within RPMD, we face a choice as to how to treat the bath degrees of freedom.

Explicit bath The most straightforward option is to discretize the bath into a finite set of harmonic oscillators. One then converges the rate constant not only with the number of ring-polymer beads, but also with the number of bath modes. This however comes with a number of disadvantages, one being that a large number of modes are needed, which increases the computational cost considerably. Additionally, including more bath modes also implies that the difference in time scale between the slowest and fastest modes grows, which in turn results in the need for shorter time steps (for the high-frequency modes) and longer simulation times (for the low-frequency modes). In this work, we used 500 modes for each bath with the discretization scheme described in Ref. 2. To ensure stable integration of high-frequency modes, we used a Cayley integration algorithm in the normal modes of all harmonic degrees of freedom, as described in Appendix A of Ref. 3. The dividing surface was chosen such that it is perpendicular to the unstable mode at the transition state.

Implicit bath It is possible to analytically integrate out the bath degrees of freedom within the ring-polymer framework. This procedure is outlined in the Appendix of Ref. 4. Static effects of the bath are captured by an effective ring-polymer Hamiltonian in the phase

space of the system only, with the bath having the effect of making the ring-polymer springs stiffer. Dynamic friction effects are captured by a Generalized Langevin Equation (GLE).

For an Ohmic bath, we can evaluate the GLE friction kernel analytically. However, as integrating the GLE is still very expensive, we choose to approximate the friction kernel by its Markovian short- and long-time limits. If simulations in both of these limits give identical results, which is usually the case, we can infer that the dynamical friction on the internal modes of the ring polymer does not play an important role, and thereby avoid having to integrate the GLE.

For describing a Debye bath, we can avoid evaluation of the friction kernel altogether by noticing that: 1) by coupling the system to a harmonic oscillator, and this harmonic oscillator in turn to an Ohmic bath, we have effectively coupled the system to a Brownian oscillator; and 2) in the limit of an overdamped oscillator, the Brownian oscillator spectral density reduces to a Debye spectral density.[5] Hence, by tuning the frequency of the added harmonic oscillator and the Ohmic friction (which we already know how to treat implicitly), we can mimic the behavior of a specific Debye bath. This approach is illustrated in Figure S1.

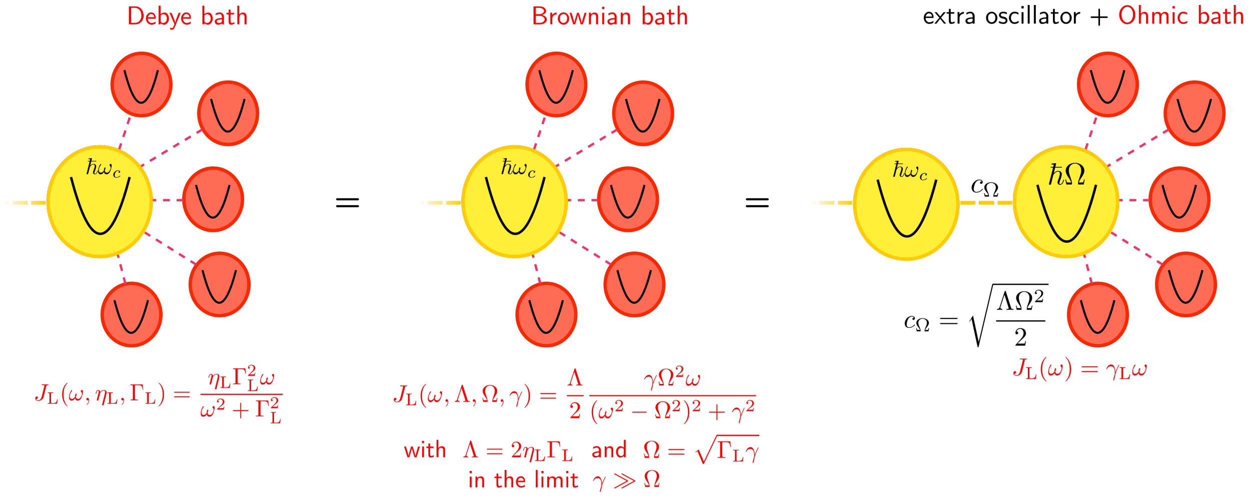

Figure S1: Schematic diagram of the relation between a Debye bath, a Brownian bath, and an extra system harmonic oscillator coupled to an Ohmic bath. Here it is illustrated for the cavity mode and its bath, but the same procedure holds for the matter part of the system. For further details, see *e.g.* Refs. 4 and 5.

We found that for describing the Debye bath coupled to the molecular coordinate $J_R(\omega)$, a harmonic oscillator with $\Omega = 2000\,\mathrm{cm}^{-1}$ coupled to an Ohmic bath with $\gamma = 20\,000\,\mathrm{cm}^{-1}$ is sufficient. For the Debye bath coupled to the cavity mode $J_\mathrm{L}(\omega)$, we need to go to $\gamma = 100\,000\,\mathrm{cm}^{-1}$, turning the harmonic oscillator's frequency up to $\Omega = 10\,000\,\mathrm{cm}^{-1}$. Due to this large frequency, we would require a much smaller time step, making the simulations more expensive. As the calculations in the low-friction regime are already very demanding, we opted for replacing the Debye bath entirely with its Ohmic equivalent (via Eq. (6) of the main text). Results supporting this replacement are presented in Section 2.

For the implicit-bath calculations, we choose the dividing surface to be in the double-well coordinate R only, *i.e.* $s(\mathbf{q}) = \sum_i R_i/N$. We evaluated $C_\mathrm{fs}(t)$ using the Bennett-Chandler approach.[6,7]

Types of bath used for the figures in the main text In this study, we used both of the approaches outlined above, as each performs well in a different regime.

For the Kramers curve for system **I** (Figure 1c of the main text), the implicit-bath approach is more computationally efficient in the low-friction regime. We therefore used this method for all calculations shown in Figure 2. For the higher friction values in Figure 1c, approximating the friction kernel by its two limits gives slightly different results; importantly, when taking the long-time limit of the friction kernel, Eq. S4 does not plateau, so the rate constant becomes ill-defined with this method. Fortunately, in this regime the explicit-bath approach becomes cheaper, so we calculated the Kramers curve in Figure 1c using this method. Note that both approaches agree for the lower friction values in this figure, where we deem our approximation to the friction kernel to be valid.

For the Kramers curve for system **II** (Figure 3b), we found that our approximation to the friction kernel remains valid for all frictions shown, the implicit- and explicit-bath methods give the same curve. For the cavity-modified rates in Figure 3c, we employed the implicit-bath approach, as it has one fewer convergence parameters (the number of bath modes).

These details are summarized in Table 1. Additionally, the most important parameters used for converging the transmission coefficient are given in Table 2.

Table 1: Summary of the types of baths used for each of the Figures in the main text.

	**bath treatment**	**double-well bath**	**cavity-mode bath**
Figure 1c	explicit*	Debye	Debye
Figure 2	implicit	Debye†	Ohmic
Figure 3b	implicit	Debye†	Ohmic
Figure 3c	implicit	Debye†	Ohmic

* It is only in this case that one needs to use an explicit bath, as the implicit-bath method breaks down; for all of the other Figures, both approaches yield the same valid results.
† For the calculations performed with an implicit bath, the Debye spectral density was effectively constructed by coupling the system to an extra oscillator, which in turn experiences Ohmic friction, as described in Figure S1.

Table 2: Summary of the most important convergence parameters used (number of beads N, and the simulation time T, time step dt and number of trajectories n used for the calculation of the transmission coefficient).

	N	T **(fs)**	dt **(fs)**	n
Figure 1c	32	1000	0.5	1×10^6
Figure 2	512	6000	0.025	4×10^5
Figure 3b	64	4500	0.015	3×10^5
Figure 3c	64	4500	0.015	2.25×10^5

2 The spectral density of the cavity mode bath: Debye vs. Ohmic

As noted in the main text, we replaced the Debye spectral density for the cavity mode used for the HEOM calculations[8] with an Ohmic spectral density, to make the computational costs more tractable. In the following, we justify this by showing that even for the strongest coupling between the cavity mode and its bath, this replacement does not change our results within the error bars. This is shown in Figure S2 for $\tau_c = 100\,\text{fs}$, $\eta_s = 0.1\omega_b$ and $\eta = 0.00125$ for system **I** (*c.f.* Figure 2 of the main text). Both classically and within RPMD, the results for a Debye bath agree well with those for an Ohmic bath, although due to the high computational cost, fewer trajectories were run such that the error bars on the RPMD results with the Debye bath remain rather large.

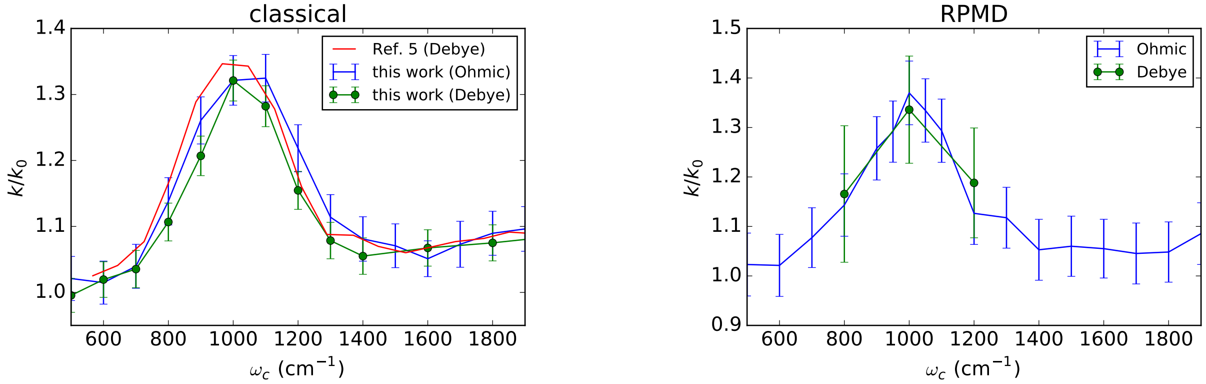

Figure S2: Comparison of the cavity-induced rate modification for the cavity coupled to an Ohmic bath vs. a Debye bath.

References

(1) Craig, I. R.; Thoss, M.; Wang, H. Proton transfer reactions in model condensed-phase environments: Accurate quantum dynamics using the multilayer multiconfiguration time-dependent Hartree approach. *J. Chem. Phys.* **2007**, *127*, 144503.

(2) Hele, T. J. H. An electronically non-adiabatic generalization of ring polymer molecular dynamics. M.Sc. thesis, University of Oxford, 2011; https://arxiv.org/abs/1308.3950.

(3) Hunt, A. C. Chemical reaction dynamics under vibrational strong coupling. M.Sc. thesis, University of Oxford, 2023.

(4) Lawrence, J. E.; Fletcher, T.; Lindoy, L. P.; Manolopoulos, D. E. On the calculation of quantum mechanical electron transfer rates. *J. Chem. Phys.* **2019**, *151*, 114119.

(5) Thoss, M.; Wang, H.; Miller, W. H. Self-consistent hybrid approach for complex systems: Application to the spin-boson model with Debye spectral density. *J. Chem. Phys.* **2001**, *115*, 2991–3005.

(6) Bennett, C. Algorithms for chemical computations. ACS Symposium Series. 1977; pp 63–97.

(7) Chandler, D. Statistical mechanics of isomerization dynamics in liquids and the transition state approximation. *J. Chem. Phys.* **1978**, *68*, 2959–2970.

(8) Lindoy, L. P.; Mandal, A.; Reichman, D. R. Quantum dynamical effects of vibrational strong coupling in chemical reactivity. *Nat. Comm.* **2023**, *14*, 2733.
